# The era of E-learning from the perspectives of Jordanian medical students: A cross-sectional study

**DOI:** 10.1016/j.heliyon.2022.e09928

**Published:** 2022-07-11

**Authors:** Muna Barakat, Rana Abu Farha, Suhaib Muflih, Ala’a B. Al-Tammemi, Bayan Othman, Yasmin Allozi, Leen Fino

**Affiliations:** a Department of Clinical Pharmacy and Therapeutics, Faculty of Pharmacy, Applied Science Private University, 11931 Amman, Jordan; b Department of Clinical Pharmacy, Faculty of Pharmacy, Jordan University of Science and Technology, Irbid 22110, Jordan; c Department of Family and Occupational Medicine, Faculty of Medicine, University of Debrecen, Debrecen, Hungary; d Doctoral School of Health Sciences, University of Debrecen, Debrecen, Hungary; e Department of Pharmaceutical Sciences and Pharmaceutics, Faculty of Pharmacy, Applied Science Private University, Amman 1931, Jordan

**Keywords:** E-learning, Medical, Students, Perception, Jordan, COVID-19

## Abstract

**Introduction:**

Online learning is becoming a crucial part of the educational process worldwide, especially after the recent COVID-19 pandemic. This study was designed to assess medical students’ perception toward online learning and their perceived preparedness and barriers during the COVID-19 pandemic.

**Methods:**

An electronic-based, cross-sectional survey was used to recruit eligible students in Pharmacy, Doctor of Pharmacy, Medicine, Nursing, Dentistry, and Veterinary Medicine programs at various Jordanian universities (public and private). Descriptive and linear regression analysis were conducted using S.P.S.S. software. The perception score was calculated based on a 5-point Likert scale.

**Results:**

A total of 939 students agreed to participate in this study. The prominent category was females (n = 691, 73.6%), the median age of students was 22.0 years (IQR = 2.0), and around 56% of the students study in private universities (n = 520, 55.6%). More than half of the students reported that their experiences were unsatisfactory or very unsatisfactory (n = 510, 54.3%). The majority of students preferred face-to-face communication with their professors and colleagues and considered it more effective (n = 682, 72.6%). The median of the mean perception score was 2.4 (IQR = 1.1). Regarding challenges and barriers, more than 70% reported weak internet connection, E-learning boredom, and lack of motivation (n = 723, 77.0%).

**Conclusion:**

This study reported inadequate satisfaction and perception towards the current experience in E-learning during the COVID-19 pandemic. It also discussed the barriers and challenges hindering this transition, such as weak internet connection and the lack of motivation, indicating a need for implementing new pedagogies to enhance students’ experiences regarding online education.

## 1 Introduction

Since the World Health Organization (WHO) declared the infectious coronavirus (COVID-19) disease a global health pandemic, substantial consequences on various aspects of life occurred, including education [1, 2, 3]. As social and physical distancing, in addition to self-quarantining, have been imposed by many governments in order to halt the spread of the virus, drastic changes in the educational system followed. Universities globally shifted toward online learning using various distance learning platforms, such as Microsoft Teams, Zoom, E-learning, Moodle and many more, enclosing new concerns and challenges for students [4, 5, 6].

Online learning is becoming a crucial part of the educational process worldwide. The concept of online learning involves implementing advanced technologies, including computers and the internet, to deliver course content, engage learners, and facilitate two-way communication between students and teachers [7]. Nevertheless, in developing countries, the lack of financial, network, and technical infrastructures such as computers and internet access challenges the implementation of distance learning [8]. On the contrary, internet services, instructors’ technical competencies, and online education experience are much more well-established in developed countries, making the process of distance learning more feasible [9, 10].

In Jordan, the concept of E-learning was introduced even before the COVID-19 era. Firstly, educational content over the internet was introduced to students during physical classroom presence. Following that, the concept of blended learning was developed, in which the participation of a teacher (face to face) and E-learning were combined together [11]. Jordan showed earlier interest in e-learning; however, it was not being utilized by most Jordanian Universities [12]. As well, with the emersion of the COVID-19 situation, the learning process has been entirely directed to online teaching due to the imposed circumstances.

Several studies were conducted to assess university students’ feedback and attitudes towards online learning [13, 14, 15]. Positive and negative aspects were reported among the student’s feedback on the online learning process. Enhanced utility of time, cost-effectiveness, comfort, accessibility and convenience were reported advantages. However, technical and behavioral challenges, content perception and instructors’ ability to use technology and provide efficient online lessons were amongst the reported barriers [13, 14, 15, 16].

Medical students’ education, practice, career progression, and mental health were affected due to COVID-19.^17^^,^ ^18^^,^ ^19^ Medical students have reported dissatisfaction and negative attitudes toward online learning, as their opportunities to learn essential practical skills were lost due to the consequences of COVID-19 ^20-22^. Therefore, this study was designed to assess medical students’ perception of online learning and their preparedness and barriers during the COVID-19 pandemic.

## 2 Methods

This study’s data are based on students’ perception of online education offered by medical schools at Jordanian universities. An electronic-based cross-sectional survey was used to recruit eligible participants in this study. Eligible participants were students in Pharmacy, Doctor of Pharmacy, Medicine, Nursing, Dentistry, and veterinary medicine programs at various Jordanian universities. From July 5th through September 22^nd^, 2021, a campaign approach using a combination of online social media and Web-based survey software was implemented to recruit the survey participants and collect data for this study.

A consent form attached online with the survey was required to be signed voluntarily by the participants before completing the study. Students who agreed to participate signed the informed consent. Participants were able to complete the survey within about 15 min. They were informed that their participation might increase their understanding of the perception towards online education. Although it was filled anonymously, the authors assured that the survey data were protected and treated with high confidentiality. The study's ethical approval was obtained from the Institutional Review Board of Applied Science Private University, Jordan [Ethical approval number: 2021-PHA-18].

### 2.1 Study tool

A structured survey was adopted and modified from previous literature [23, 24, 25], utilizing the general principles of good survey design. The survey was distributed to participants in both Arabic and English versions using Google Form platform (A web-based survey tool provided by Google®). After the initial development of the survey, it was evaluated by a team of 15 experts in educational technology and socio-behavioral sciences to assess the face and content validity of the survey items. The questions were made free of medical jargon or difficult terminology. The questionnaire was developed in English, then was translated into Arabic using translation and back-translation techniques by two independent academic translators. Also, to ensure clarity, readability, and understandability, the questionnaire was piloted (in both languages) on 15 students, and refinements were made as needed.

The survey consists of a total of 3434 questions distributed over four parts, including sociodemographic data for the students (9 questions), students’ perceptions towards online learning (1414 statements), students’ perceptions towards the obstacles surrounding the E-learning (99 statements), and students’ experience with E-learning tools and their satisfaction (2 Questions). Using Cronbach’s alphas, the reliability of the scales was determined, and it showed that the alpha for the perception Likert scale questions was 0.81, which indicated that the items would form a scale of high internal consistency. Mean perception score was calculated based on a 5-point Likert scale (5: strongly agree, 4: agree, 3: neutral, 2: disagree, and 1: strongly disagree) for the positive perception statements and vice versa for the negative statements (5: strongly disagree, 4: disagree, 3: neutral, 2: Agree, and 1: strongly agree). The higher mean perception score indicates a better perception towards E-learning.

### 2.2 Statistical analysis

Study data were extracted from an excel sheet obtained from the google form platform. It was converted and analysed using I.B.M. statistical package for social sciences (IBM SPSS Statistics, version 22.0, Chicago, Illinois). Descriptive analyses were presented as median ± interquartile range (IQR) for continuous variables, while frequency and proportions were used for categorical variables.

Simple linear regression was carried out to initially screen the independent variables affecting students’ perception of E-learning. Variables with P-value< 0.25 using univariate linear regression analysis were entered into multiple linear regression analysis. Variables were selected after checking their independence, where person correlation <0.9 indicates the absence of multicollinearity between the independent variables in regression analysis. In the multiple linear regression analysis, variables that were independently affecting the perception of E-learning were identified. A P-value of ≤0.05 was considered statistically significant.

## 3 Results

### 3.1 Sociodemographic characteristics of the study participants

During the study period, 939 students agreed to take part in this study and filled out the study questionnaire. Around three-quarters of the students were females (n = 691, 73.6%). Students have a median age of 22.0 years (IQR = 2.0), and around 56% of them are studying in private universities (n = 520, 55.6%). The most common major among the recruited sample was a bachelor of pharmacy (n = 664, 70.7%). The majority of students reside in urban areas (795, 84.7%), and only 25.6% (n = 240) reported that they had been infected previously with the coronavirus. The median number of hours spent by students online for non-educational purposes was 14.0 h per week (IQR = 24.0), while they spent a median of 19.0 h per week for educational purposes (IQR = 22.0). Demographic characteristics are presented in Table 1.

**Table 1:** Demographic characteristics of the study participants (n = 939).

Parameter	Median (IQR)	n (%)
Age (years)	21.0 (2.0)	
Gender		
• Female		691 (73.6)
• Male		248 (26.4)
Major		
• Bachelor of Pharmacy		664 (70.7)
• Doctor of Pharmacy		49 (5.2)
• Medicine		180 (19.2)
• Nursing		29 (3.1)
• Dentistry		17 (1.8)
Years of study		
• First and second year		361 (38.4)
• Third and fourth year		383 (40.8)
• ≥Fifth year		195 (20.8)
University		
• Private		520 (55.6)
• Public		419 (44.6)
Residential area		
• Urban		795 (84.7)
• Rural		144 (15.3)
Have you been infected with the corona virus?		
• No		515 (54.8)
• Yes		240 (25.6)
• Unsure		184 (19.6)
The number of hours you spend online per week for non-educational purposes	14.0 (24.0)	
The number of hours you spend online per week for educational purposes	19.0 (22.0)	

IQR: interquartile range.

### 3.2 Students’ satisfaction with the current experience in E-learning during the COVID-19 pandemic

**Figure 1:**
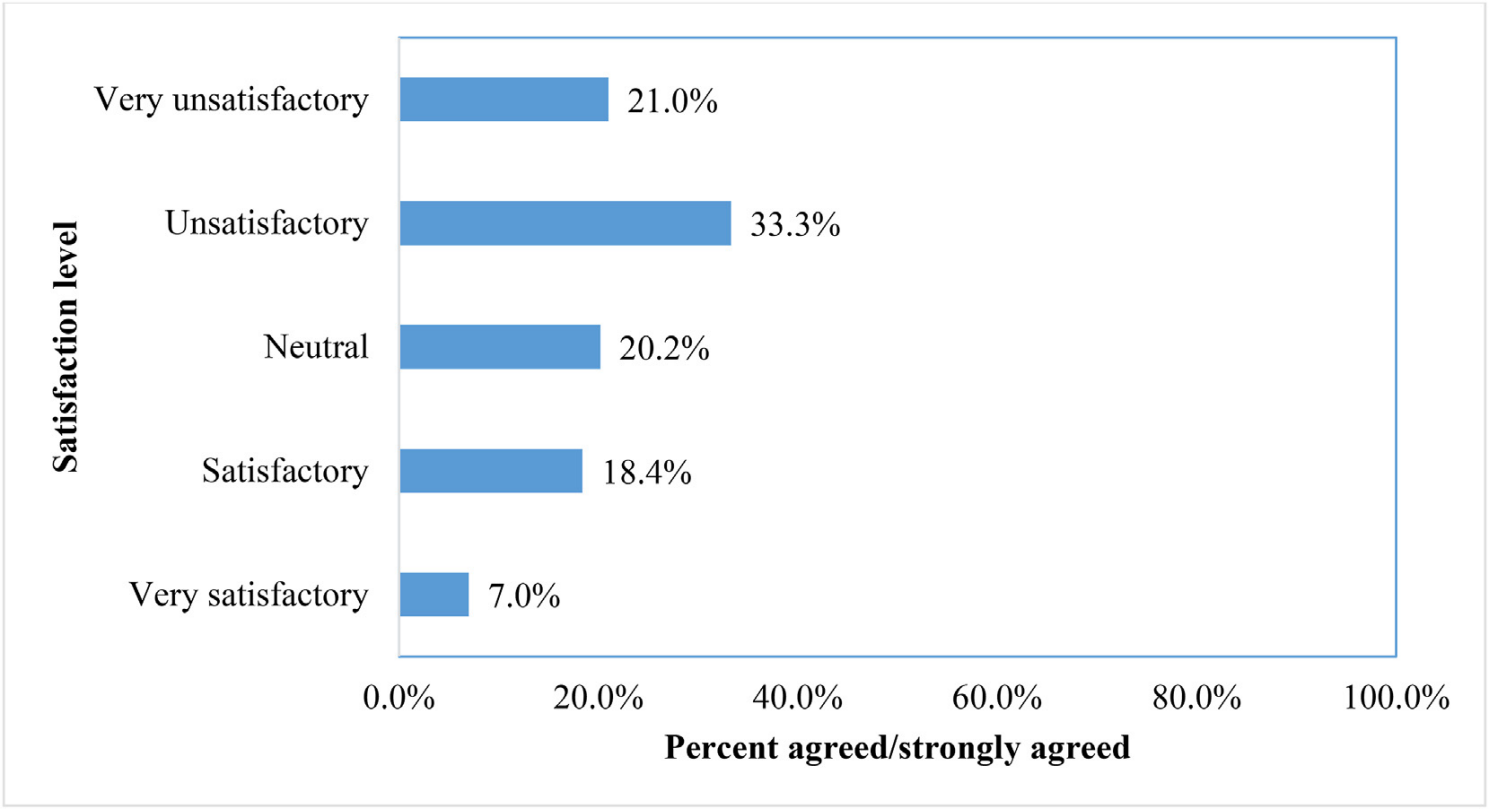
Students' satisfaction with the current experience in E-learning during the COVID-19 pandemic (n = 939).

Students reported inadequate satisfaction with their current experience in E-learning during the COVID-19 pandemic (n = 939) (Figure 1), where only 7.0% of them (n = 66) reported that their experiences with E-learning were very satisfactory, while 18.4% of them (n = 173) reported that their experiences were satisfactory.

### 3.3 Students’ perception towards E-learning experience during the COVID-19 pandemic

Students also showed inadequate perception towards their E-learning experience (Table 2), where only 24.7% of them (n = 232) agreed/strongly agreed that E-learning helps them achieve their future plans. Also, only 16.2% of the students (n = 152) prefer E-learning to become the new normal. Moreover, only 16.8% of them (n = 157) feel that studying the courses online will help them to memorize and master them better. In addition, only one-third of the students believed that their universities deliver a high-quality online learning experience (n = 321, 34.2%) and provide technical support for E-learning (n = 313, 33.3%). Also, the mean perception score was 2.4 (IQR = 1.1).

**Table 2:** Students' perception towards E-learning experience during COVID-19 pandemic (n = 939).

Statements	Strongly agree/Agree	Neutral	Strongly disagree/Disagree
E-learning helps me achieve my future plans (travel, get a higher degree, etc.)	232 (24.7)	201 (21.4)	506 (53.9)
In general, my university delivers a high-quality online learning experience	321 (34.2)	279 (29.7)	339 (36.1)
I would prefer e-learning to become the new normal	152 (16.2)	135 (14.4)	652 (69.4)
I feel comfortable communicating with my professors and colleagues electronically	221 (23.5)	178 (19.0)	540 (57.5)
I feel that studying the courses online will help me to memorize and master them better	157 (16.8)	159 (16.9)	623 (66.3)
Electronic courses help to organize study time and perform academic tasks better than university face-face education	254 (27.1)	167 (17.8)	518 (55.2)
I have satisfactory computer skills for dealing with online courses/assignments	477 (50.8)	204 (21.7)	258 (27.4)
I can ask questions and get teachers' answers quickly electronically	265 (28.2)	32 (29.4)	406 (43.2)
I prefer face-to-face communication with my professors and colleagues because it is more effective	682 (72.6)	122 (13.0)	135 (14.4)
I can easily work in a group in electronic courses	259 (27.6)	264 (28.1)	416 (44.3)
All my courses can be taken electronically without difficulties	192 (20.4)	139 (14.8)	608 (64.7)
My university provides technical support for e-learning	313 (33.3)	290 (30.9)	336 (35.8)
E-learning leads to an educational overload on students	487 (51.9)	160 (17.0)	192 (20.4)
E-learning helps brainstorm better ideas than classroom study	164 (64.2)	205 (17.5)	570 (60.7)

### 3.4 Students' perceptions towards the obstacles surrounding the E-learning

The obstacles surrounding E-learning were assessed using 9 statements (Table 3). Weak internet connection (n = 755, 80.4%) was the most perceived obstacle, followed by the boredom of E-learning (n = 731, 77.8%), home related conditions (n = 729, 77.6%), and the lack of motivation (n = 723, 77.0%).

**Table 3:** Students' perceptions towards the obstacles surrounding the E-learning (n = 939).

Statements	Strongly agree/Agree	Neutral	Strongly disagree/Disagree
Lack of motivation	723 (77.0)	100 (10.6)	116 (12.4)
Lack of instructions	648 (69.0)	168 (17.9)	123 (13.1)
Difficulty in dealing with electronic teaching tools	433 (46.1)	230 (24.5)	276 (29.4)
Cost of equipment for e-learning (computer, headphones, etc.)	629 (67.0)	163 (14.7)	147 (15.7)
Internet subscription cost	619 (65.9)	181 (19.3)	139 (14.8)
Home-related conditions	729 (77.6)	118 (12.6)	92 (9.8)
Weak internet connection	755 (80.4)	100 (10.6)	84 (8.9)
Too much time consuming	674 (71.8)	135 (14.4)	130 (13.8)
E-learning is boring	731 (77.8)	95 (10.1)	113 (12.0)

### 3.5 Students’ experience with E-learning tools

**Figure 2:**
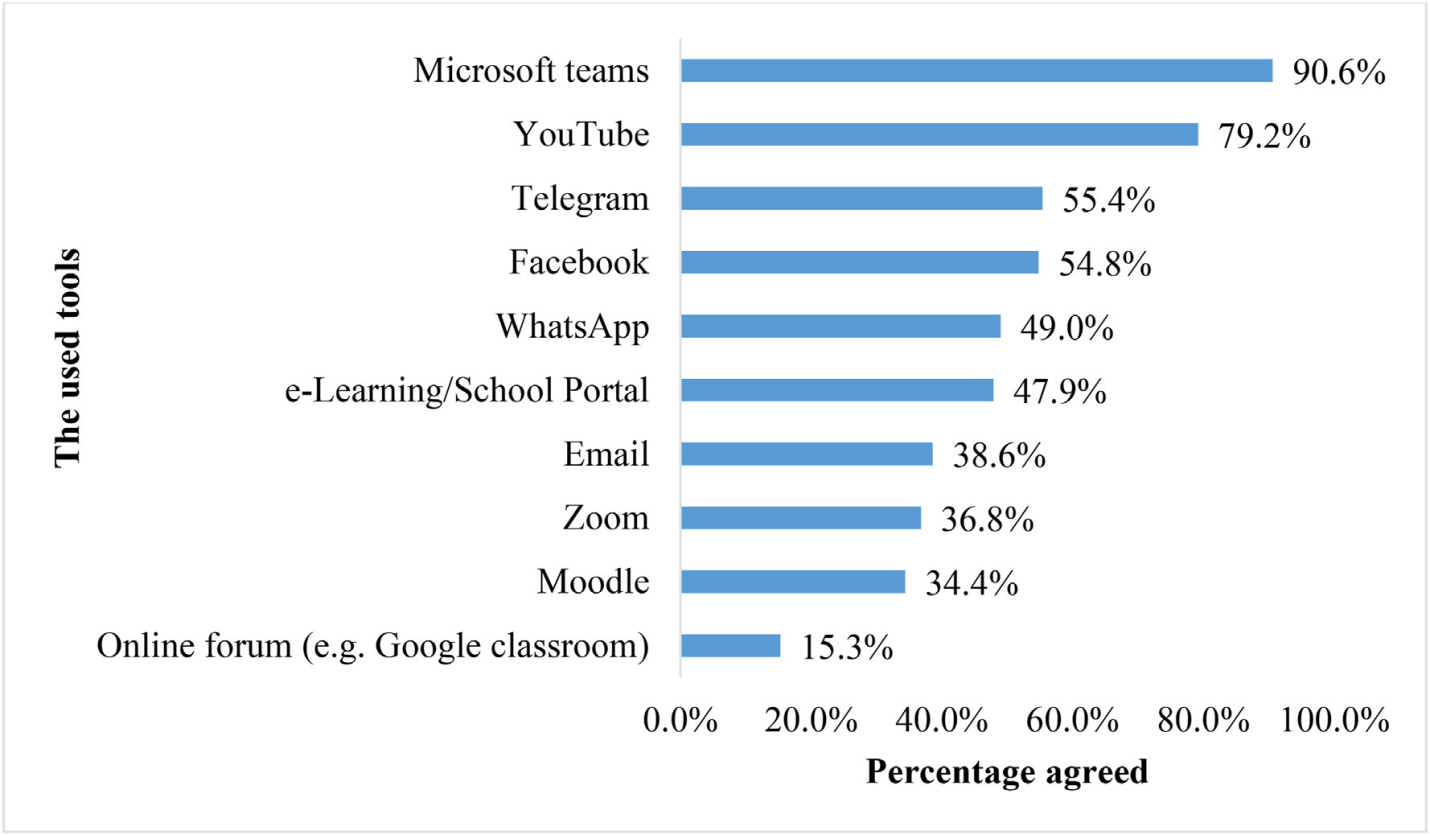
. Students' experience with E-learning tools (n = 939).

Students were asked about the tools they used during their online education (Figure 2). Microsoft teams was the most commonly used tool (n = 851, 90.6%), followed by YouTube (n = 744, 79.2%) and Telegram (n = 520, 55.4%), while Goggle Classroom was the least used tool (n = 144, 15.3%).

### 3.6 Assessment of factors affecting students’ perception of E-learning

Lastly, univariate and multivariate linear regression analyses were performed to evaluate factors affecting the perception of E-learning (Table 4). Results showed that those of higher age, male students, and those from private universities showed a better perception of E-learning than others (P ≤ 0.05).

**Table 4:** Assessment of factors affecting students' perception towards E-learning (n = 939).

Parameter	Perception score			
	Beta	P-value^b^	Beta	P-value^c^
Age (years)	0.202	<0.001^a^	0.139	<0.001^d^
Gender				
• Female	Reference			
• Male	0.143	<0.001^a^	0.100	0.001^d^
Years of study				
• First and second year	Reference			
• Third and fourth year	0.069	0.035^a^	0.026	0.419
• ≥Fifth year	0.099	0.002^a^	0.039	0.320
University				
• Public	Reference			
• Private	0.146	<0.001^a^	0.102	0.003^d^
Residential area				
• Urban	Reference			
• Rural	-0.046	0.160^a^	0.018	0.592
Have you been infected with the corona virus?				
• No/unsure	Reference			
• Yes	-0.032	0.326	—	—
The number of hours you spend online per week for non-educational purposes	-0.064	0.023^a^	-0.063	0.288
The number of hours you spend online per week for educational purposes	-0.053	0.107^a^	0.004	0.941

a Eligible for entry in multiple linear regression

b Using simple linear regression

c Using multiple linear regression

d Significant at 0.05 significance level.

## 4 Discussion

This study was designed to assess medical students’ perception and preparedness in both public and private universities around Jordan toward the unprecedented COVID-19 pandemic mandated shifts in educational approaches. It also aimed to explore the barriers and challenges encountered by students with the transition to online learning platforms. Findings of this investigation indicated a general student dissatisfaction with their current experience with E-learning.

Participants of this study demonstrated an overall negative perception of E-learning, preferring the face-to-face teaching approach, which facilitates more traditional interaction methods with colleagues and educators. This was in line with numerous studies conducted in both developing and advanced economies [16, 20, 26, 27, 28]. Participants in Al Balas *et al.’s* study, for example, considered ‘distance education’ a chief obstacle in acquiring adequate clinical skills in their degrees [16]. Alrashhed *et al.* suggested incorporating an interprofessional education (IPE) virtual experience during the COVID-19 pandemic, as it was found that it improves student engagement and interaction amongst students themselves as well as with their mentors [29]. Such adoption requires the employment of numerous teaching and learning approaches, including asynchronized discussions, synchronized teaching with breakup sessions, and case-based assignments [29].

[16] Another study conducted on one public university in Jordan, including only medical students, revealed that 75% of its participants were not satisfied with their experience with online learning [22]. Students in such studies often discussed integrating online teaching methods with traditional pedagogies in university education [9, 22]. Moreover, similar results were observed in a study that surveyed undergraduate university students in the United States. This study aimed to grasp students’ experiences regarding the transitioning of undergraduate education from a remote lectures approach due to the COVID-19 outbreak. The participants negatively perceived this transition and deemed the transitioned courses to become “less enjoyable and less interesting [30]”.

The conferred negative perception towards the shift to online E-learning is arguably rationalized by the emergency crisis response to the COVID-19 pandemic, with the unprecedented challenges triggered by it and its concomitant quarantine to the unfamiliarity with the online experience [26]. This impacted students’ perceptions concerning the online learning experience. Universities had to abruptly adapt to the new situation, implementing methods that are often limited and lacking the framework of effective online education theories and pedagogies [31, 32].

Furthermore, online E-learning components are inherently technology-driven, relying on internet facilities and educational institutions’ collaborations with telecommunication industries [31]. Jordan is considered one of the developing countries, recognizing the fact that a “developing country” or an “evolving economy [33, 34]”, which are terms commonly employed to refer to a population with a low level of material well-being, where poor information technology infrastructure, lack of financial resources and technical support, are considered of main challenges for online distance learning transition [9, 33, 34, 35, 36]. Findings of this study highlighted that weak internet connection issues were perceived as a major obstacle encountering students. Not to mention that throughout the current Covid-19 pandemic, reports demonstrate increased poverty levels in communities, affecting internet accessibility amongst affected individuals. Consequently, students with such low socioeconomic status had little or no access to broadband connections, thus affecting their performance and attitude regarding E-learning [31]. Interestingly, this study underlined the influence of the variation in socioeconomic status, as private university students demonstrated a significantly more positive perception towards E-learning compared to others - given that students enrolled in private universities often have a higher socioeconomic status [37].

Furthermore, Jordan telecommunication companies had suffered from an overwhelming load on the internet network resulting in a diminished internet speed and connection in numerous areas [22]. Encountering such experiences whilst attending classes, taking exams, or even submitting assignments, is understandably associated with feelings of helplessness and contributes to students’ overall dissatisfaction with the online experience [21].

Another major obstacle affecting students’ perception of online learning was found to be home-related issues. This was in line with findings of one qualitative study conducted in Jordan around nursing students’ experiences during the national curfew mandated by the Jordanian government and the following imposed order by the Ministry of Higher Education locking down the academic institutions [21, 38]. This qualitative research reported that female students with children were struggling to manage daily schedules at home and described having time management difficulties; some even discussed the lack of support provided by their partners [21]. Evidence from literature demonstrated the negative effect of distanced E-learning on students’ mental health, revealing that some students experienced feeling helpless, burdened, and suffering from burnout symptoms [21, 39, 40]. Moreover, boredom and lack of motivation were also challenges encountered by participants of this study. Such obstacles can be elucidated by reasons such as students’ lack of interest and the teaching approaches used. The traditional teaching model often requires students to attend monotonous lectures lacking visual stimulation with little opportunity for students to engage in discussions. This teaching mode often results in students 'absenteeism' (i.e., feeling less motivated to attend future lectures) [41]. Such aspects might be compensated by the inclusion of video-conferencing which is believed to enhance users’ interaction and their overall experience [41, 42].

This study also highlighted the importance of incorporating a deeper and more rigorous effort in adapting new teaching approaches to help overcome the obstacles hindering effective online transition [32]. Such changes may include training staff members in teaching pedagogies to improve the quality and delivery of lectures [41]. Additionally, training students and staff members to acquaint them with the online learning tools might be of value, as studies underlined the lack of familiarity with synchronous tools among learners who were accustomed to asynchronous approaches to online learning (e.g., Zoom and Microsoft Teams) [16, 30, 43, 44]. It also might be essential for universities to adopt new information technologies and services to facilitate learning processes [44, 45, 46]. Subsidizing internet subscriptions' costs for students and instructors might be of help [31].

### 4.1 Limitations

Several limitations can be identified for this study. First, because the survey used in this study was distributed online, particularly through social media (e.g., Facebook), students who are not active social media users or have poor internet connections were unable to participate, potentially leading to bias in terms of who could participate. Second, by using the online survey, it was hard to know the number of students who received the survey to calculate the response rate. Third, the survey contained self-reported information that recall bias could have influenced. Furthermore, using an online survey rather than a face-to-face meeting puts the study data's trustworthiness and authenticity in danger. However, considering the COVID-19 pandemic's limitation measures, this methodology was the best alternative. Fourth, the survey did not include specific questions related to online learning of medicine/pharmacology, which must be considered in future studies. Finally, as the sample recruitment was not random, using the snowball collection technique, selection bias could be an issue. Nonetheless, our research has provided baseline data on E-learning in Jordanian students, which may aid in better education patterns and stimulate more research on this growing topic.

## 5 Conclusions

As students reported inadequate satisfaction and perception towards the current experience in E-learning during the COVID-19 pandemic. This study highlighted medical students' perception and preparedness toward the COVID-19 mandated shifts in educational approaches. It also discussed the barriers and challenges hindering this transition, such as weak internet connection and the lack of motivation, indicating a need for implementing new pedagogies to enhance students' experiences regarding online education.

## Declarations

### Author contribution statement

Muna Barakat; Rana Abu Farha; Suhaib Muflih; Ala’a B. Al-Tammemi; Bayan Othman; Yasmin Allozi; Leen Fino: Conceived and designed the experiments; Performed the experiments; Analyzed and interpreted the data; Contributed reagents, materials, analysis tools or data; Wrote the paper.

### Funding statement

This research did not receive any specific grant from funding agencies in the public, commercial, or not-for-profit sectors.

### Data availability statement

Data will be made available on request.

### Declaration of interest’s statement

The authors declare no conflict of interest.

### Additional information

Supplementary content related to this article has been published online at https://doi.org/10.1016/j.heliyon.2022.e09928.

## Appendix ASupplementary data

Questionnaire Questionnaire
