# Supplementary material for: The era of E-learning from the perspectives of Jordanian medical students: A cross-sectional study
Source: Heliyon. 2022 Jul 11;8(7):e09928. doi: 10.1016/j.heliyon.2022.e09928 (PMC9304731; doi:10.1016/j.heliyon.2022.e09928)
Supplement: Questionnaire [file mmc1.docx]

**The Era of E-Learning from the students’ perspectives: A cross-sectional study**

**Part 1. Sociodemographic Data for the Students**

1. **Gender**

- Male
- Female

1. **Age (Years)……………………….**
2. **Major**

- Bachelor of Pharmacy
- Pharm.D.
- Medicine
- Nurse
- Dentist

1. **Years of Program**

- 1st (freshmen)
- 2nd
- 3rd
- 4th
- 5th
- 6th

1. **Type of University**

- Public
- Private

1. **Type of living area**

- Urban
- Rural

1. **Have you been infected with Corona virus?**

- Yes
- No
- Not sure

1. **Indicate the number of hours you generally spend per week online for non- educational purposes (e.g., Social media) ……………………………………………………………**
2. **Indicate the number of hours you generally spend per week online for educational purposes during COVID-19 pandemic ……………………………………………….**

**Part2. Students' perceptions towards online learning**

1. **For the following statements, choose the level of agreement with each one:**

| **Statement** | **Strongly Agree** | **Agree** | **Neutral** | **Disagree** | **Strongly Disagree** |
| --- | --- | --- | --- | --- | --- |
| E-learning helps me achieve my future plans (travel, get a higher degree, etc.) |  |  |  |  |  |
| In general, my university delivers a high-quality online learning experience |  |  |  |  |  |
| I would prefer e-learning to become the new normal |  |  |  |  |  |
| I feel comfortable communicating with my professors and colleagues electronically |  |  |  |  |  |
| I feel that studying the courses online will help me to memorize and master them better |  |  |  |  |  |
| Electronic courses help to organize study time and perform academic tasks better than university face-face education |  |  |  |  |  |
| I have satisfactory computer skills for dealing with online courses/assignments |  |  |  |  |  |
| I can ask questions and get teachers' answers quickly electronically |  |  |  |  |  |
| I prefer face-to-face communication with my professors and colleagues because it is more effective |  |  |  |  |  |
| I can easily work in a group in electronic courses |  |  |  |  |  |
| All my courses can be taken electronically without difficulties |  |  |  |  |  |
| My university provides technical support for e-learning |  |  |  |  |  |
| E-learning leads to an educational overload on students |  |  |  |  |  |
| E-learning helps brainstorm better ideas than classroom study |  |  |  |  |  |

**Part3. Students' perceptions towards the obstacles surrounding the e-learning**

1. **For the following Obstacles surrounding e-learning, choose the level of agreement with each one:**

| **Obstacles** | **Strongly Agree** | **Agree** | **Neutral** | **Disagree** | **Strongly Disagree** |
| --- | --- | --- | --- | --- | --- |
| Lack of motivation |  |  |  |  |  |
| Lack of instructions |  |  |  |  |  |
| Difficulty in dealing with electronic teaching tools |  |  |  |  |  |
| Cost of equipment for e-learning (computer, headphones, etc.) |  |  |  |  |  |
| Internet subscription cost |  |  |  |  |  |
| Home-related conditions |  |  |  |  |  |
| Weak internet connection |  |  |  |  |  |
| Too much time consuming |  |  |  |  |  |
| E-learning is boring |  |  |  |  |  |

**Part4. Students' experience with e-learning tools**

1. **Which of the following online tools you were using?**

| **Tools** | **Yes** | **No** |
| --- | --- | --- |
| Zoom |  |  |
| e-Learning/School Portal |  |  |
| Moodle |  |  |
| Facebook |  |  |
| YouTube |  |  |
| WhatsApp |  |  |
| Telegram |  |  |
| Email |  |  |
| Online forum (e.g. Google classroom) |  |  |
| Microsoft teams |  |  |

1. **In general, how do you evaluate the current experience in distance education using the Internet or e-learning programs in light of the COVID-19 pandemic?**

- Very satisfactory
- Satisfactory
- Neutral
- Unsatisfactory
- Very unsatisfactory
